# MUSCLE QUALITY AND REACTIVE BALANCE CONTROL IN OLDER ADULTS

**DOI:** 10.1093/geroni/igae098.2718

**Published:** 2024-12-31

**Authors:** Justin Whitten, Bryant O’Leary, Dawn Tarabochia, David Graham

**Affiliations:** Montana State University, Montana, United States; Montana State University, Bozeman, Montana, United States; Montana State University, Bozeman, Montana, United States; Griffith University, Gold Coast, Queensland, Australia

## Abstract

Falls are a predominant cause of morbidity and mortality in older adults. Muscle quality is associated with mobility and fall risk in older adults. Echo intensity (EI), obtained via ultrasonography, offers a method to quantify muscular fat infiltration and has become increasingly popular as an objective measure of morphological muscle quality. This study aimed to determine the relationship between lower limb EI and reactive balance control (RBC) in community-dwelling older adults. We analyzed EI and muscle thickness in the biceps femoris (BF), vastus lateralis (VL), and medial gastrocnemius (MG) across 18 participants (12 older adults, age: 65 ± 4.1; 6 younger controls, age: 25 ± 3.4) before administering specific assessments of RBC including the Choice Stepping Reaction Test and the Lean and Release Test. BF and MG EI were associated with LRT performance (t=3.112, p<0.001; t=-3.597, p<0.01), and BF EI was associated with CSRT performance (t=-4.281, p<0.001). However, these associations were characterized by small effect sizes (d=-0.17, d=-0.19 and, d=0.12 respectively) and were not significant after controlling for age. Although muscle quality is important for functional mobility it was not associated with RBC in the present study. This is supported by previous research that balance recovery appears to depend on the ability to generate efficiently coordinated neuromotor responses rather than on general qualities such as muscle force or muscle quality and thickness alone and underscores the importance of specific training to improve balance recovery. Further research, with larger sample sizes, is needed to understand the influence of EI on RBC.

